# Anti-SARS-CoV-2 Activity of Adamantanes In Vitro and in Animal Models of Infection

**DOI:** 10.3390/covid2110111

**Published:** 2022-10-28

**Authors:** Sun-Young Lim, Zhiru Guo, Ping Liu, Lindsay G. A. McKay, Nadia Storm, Anthony Griffiths, Ming Da Qu, Robert W. Finberg, Mohan Somasundaran, Jennifer P. Wang

**Affiliations:** 1 Department of Medicine, University of Massachusetts Chan Medical School, Worcester, MA 01655, USA; 2 Department of Microbiology and National Emerging Infectious Diseases Laboratories, Boston University School of Medicine, Boston, MA 02115, USA; 3 Division of Infectious Disease & Immunology, Department of Medicine, University of Massachusetts Chan Medical School, Worcester, MA 01655, USA; 4 Department of Biochemistry and Molecular Biotechnology, University of Massachusetts Chan Medical School, Worcester, MA 01655, USA

**Keywords:** SARS-CoV-2, amantadine, rimantadine, COVID-19, hamster, ACE2-A549 cells

## Abstract

Coronavirus disease 2019 (COVID-19) has had devastating effects worldwide, with particularly high morbidity and mortality in outbreaks on residential care facilities. Amantadine, originally licensed as an antiviral agent for therapy and prophylaxis against influenza A virus, has beneficial effects on patients with Parkinson’s disease and is used for treatment of Parkinson’s disease, multiple sclerosis, acquired brain injury, and various other neurological disorders. Recent observational data suggest an inverse relationship between the use of amantadine and COVID-19. Adamantanes, including amantadine and rimantadine, are reported to have in vitro activity against severe acute respiratory syndrome coronavirus (SARS-CoV) and, more recently, SARS-CoV-2. We hypothesized that adamantanes have antiviral activity against SARS-CoV-2, including variant strains. To assess the activity of adamantanes against SARS-CoV-2, we used in vitro and in vivo models of infection. We established that amantadine, rimantadine, and tromantadine inhibit the growth of SARS-CoV-2 in vitro in cultured human epithelial cells. While neither rimantadine nor amantadine reduces lung viral titers in mice infected with mouse-adapted SARS-CoV-2, rimantadine significantly reduces viral titers in the lungs in golden Syrian hamsters infected with SARS-CoV-2. In summary, rimantadine has antiviral activity against SARS-CoV-2 in human alveolar epithelial cells and in the hamster model of SARS-CoV-2 lung infection. The evaluation of amantadine or rimantadine in human randomized controlled trials can definitively address applications for the treatment or prevention of COVID-19.

## 1. Introduction

The ongoing coronavirus disease 2019 (COVID-19) pandemic is caused by severe acute respiratory syndrome coronavirus 2 (SARS-CoV-2) infection, which can result in a broad range of symptoms including fever and difficulty breathing. The high transmissibility of the virus and severe outcomes of the disease has resulted in numerous fatal cases as well as economic hardships across the world. The emergence of infectious variants such as omicron has caused surges in newly confirmed cases per day, underscoring the need for accessible therapeutic treatments for numerous patients. Food and Drug Administration (FDA)-approved antiviral therapeutics for COVID-19 include the oral agents molnupiravir (EIDD-2801) and nirmatrelvir, and intravenous remdesivir. Repurposing known antivirals with activity against SARS-CoV-2 would provide for much-needed SARS-CoV-2 treatment options worldwide.

Amantadine is an antiviral approved by the FDA in 1976 for symptomatic and/or prophylactic treatment of influenza A virus (IAV) in adults. Amantadine is thought to block the early stage of IAV replication, specifically in the acidified endosome for which proton channels are formed by the M2 protein. Amantadine crosses the endosome membrane and interrupts the release of the virion into the cell. However, it is no longer recommended for the treatment or prophylaxis of IAV due to high resistance rates [1]. Amantadine has been used anecdotally for the prevention and treatment of symptomatic COVID-19 [2]. Despite close contact with SARS-CoV-2-infected people, individuals who take amantadine regularly for neurologic conditions such as parkinsonian movement disorders and post-traumatic brain injury may be protected from infection [3] and clinical disease [4]. A recent hospital-based cohort study reported that neurologic patients receiving amantadine had a reduced risk of COVID-19 [5]. Antiviral effects against human coronaviruses have been shown for other adamantane derivatives, including an amantadine analog for human coronavirus 229E [6], memantine for human coronavirus OC43 [7], and rimantadine and bananin for SARS-CoV-1 [8,9].

Given this, we tested amantadine hydrochloride and other adamantanes against SARS-CoV-2 in human epithelial cells in vitro and in two animal models in vivo: BALB/c mice and golden Syrian hamsters. We show that adamantanes have antiviral activity against SARS-CoV-2. Our collective findings underscore the importance for evaluating adamantanes in human randomized controlled trials to definitively determine their efficacy in treatment or prevention of COVID-19.

## 2. Materials and Methods

### 2.1. Cells and Viruses

Vero E6 cells (ATCC, Cat#CRL-1586, Manassas, VA, USA) were maintained in Modified Eagle’s Medium (MEM) supplemented with 10% fetal bovine serum (FBS). Human ACE2-overexpressing A549 cells (ACE2-A549), a gift from the tenOever laboratory [10], were maintained in Dulbecco’s Modified Eagle’s Medium (DMEM) supplemented with 10% FBS. The following SARS-CoV-2 strains were expanded in Vero E6 cells: infectious-clone-derived mNeonGreen SARS-CoV-2 (icSARS-CoV-2 mNG), a kind gift from Dr. Pei-Yong Shi [11]; USA-WA1/2020 (WA/01, BEI Resources Cat #NR-52281, Manassas, VA, USA); and MA10 (BEI Resources Cat #NR-55329). SARS-CoV-2 strain B.1.1.529 omicron (BEI Resources Cat #NR-56461) was expanded in Vero E6-TMPRSS2-T2A-ACE2 cells (BEI Resources Cat #NR-54970). Influenza A/Hong Kong/8/68 (H3N2) was originally from Charles River Laboratories (Wilmington, MA, USA) and subsequently grown in Madin–Darby canine kidney (MDCK) cells (ATCC Cat #NBL-2) in MEM supplemented with 1% bovine serum albumin and 10 μg/mL acetylated trypsin (Sigma, St. Louis, MO, USA).

### 2.2. Antiviral Compounds

Amantadine chloride (Millipore Sigma, Cat #1018505) and rimantadine hydrochloride (Millipore Sigma, Cat #1604508) were used for in vitro and in vivo experiments. For in vitro assays, amantadine chloride was dissolved in MEM, while rimantadine chloride was dissolved in dimethyl sulfoxide (DMSO, Fisher Scientific Cat #BP231–100, Pittsburgh, PA, USA). Tromantadine hydrochloride (MedChem Express Cat #HY-U00124, Monmouth Junction, NJ, USA), EIDD-1931 (MedChemExpress Cat #HY-125033) and remdesivir (Fisher Scientific Cat #AC469411000) were each dissolved in DMSO. For in vivo experiments, both amantadine chloride and rimantadine chloride were dissolved in water; tromantadine hydrochloride, EIDD-1931, and remdesivir were dissolved in DMSO. Pharmaceutical-grade remdesivir/cyclodextrin (VEKLURY™) was purchased from the University of Massachusetts Memorial Medical Center pharmacy and dissolved in water.

### 2.3. Antiviral Assays

ACE2-A549 cells were seeded in 96-well black polystyrene microplates (Corning Cat #3686, Oneonta, NY, USA) at 20,000 cells per well. Vero E6 cells and Vero E6-TMPRSS2-T2A-ACE2 (Vero E6 T/A) cells were seeded at 15,000 cells/well. Cells were washed twice with PBS. Viruses were diluted to appropriate concentrations and were placed on cells, mixed with MEM/2% FBS containing specific compounds. After 1 h of incubation in 37 °C with 5% CO_2_, the virus and compound mixture were removed and washed two times with PBS. The cells were replenished with fresh MEM/2% FBS with compounds. The cells were incubated for 2–3 days (depending on the cell line) at 37 °C with 5% CO_2_. After the incubation period, the supernatants were collected for virus titer quantification by plaque assay. The cells were washed with PBS and fixed with 4% paraformaldehyde (PFA), subsequently dehydrated with ethanol, and then transported out of the BSL-3 facility. Cells were rehydrated with diluted ethanol and PBS.

### 2.4. Immunostaining Assay

The rehydrated cells were stained with anti-SARS-CoV-2 spike antibody CR3022 from MassBiologics [12] or with anti-SARS-CoV-2 nucleocapsid antibody (Sinobiological Cat #40143-MM05, Wayne, PA, USA) conjugated with AlexaFluor-488 at 1:200 for 1 h at room temperature. Once the staining was complete, the cells were washed with PBS, and then, the cells were stained with 4′,6-diamidino-2-phenylindole (DAPI) (1 μg/mL, Abcam, Boston, MA, USA) for 15 min to visualize cell nuclei. Fluorescence from the cells was detected using the ImageXpress^®^ Micro-XL (IXM) Automated Imaging System. The IXM software was used to count the total numbers of AlexaFluor-488-positive cells and DAPI-positive cells. The same protocol was applied for IAV staining, using an anti-IAV nucleocapsid antibody (AbCam Cat #ab20343, Boston, MA, USA) and goat anti-mouse antibody conjugated with AlexaFluor-488 (Invitrogen Cat #A11029, Waltham, MA, USA). The images were processed by MetaXpress Software.

### 2.5. SARS-CoV-2 Plaque Assays

Vero E6 cells were seeded in 12-well plates at 80% confluency. Virus-containing supernatants were serially diluted and added to Vero E6 cell monolayers. Following 1 h adsorption, supernatants were removed, and 1× MEM with 0.7% agar was placed in each well and was allowed to solidify before being placed in 37 °C incubators. After 3 days, 4% PFA was placed on the agar overlay overnight to inactivate SARS-CoV-2. The agar overlay was removed, and the cell monolayer was washed with PBS once; 1% crystal violet dissolved in 20% ethanol was used to dye the remaining monolayer. Plaques were counted from each well and averaged to calculate the plaque forming units (PFU) per mL.

### 2.6. Viability Assay

Viability was assessed using the CellTiter-Glo^®^ Luminescent Cell Viability Assay (Promega Cat# G9242, Madison, WI, USA).

### 2.7. Animal Antiviral Studies

The mouse infection studies were performed in an animal biosafety level 3 (ABSL-3) facility at UMass Chan Medical School (UMass Chan). The study procedures were conducted with approval by the Institutional Animal Care and Use Committee at UMass Chan (PROTO201900346). Twelve-week-old female BALB/c mice (purchased from the Jackson Laboratory) were administered drugs dissolved in water by intraperitoneal (i.p.) or intranasal (i.n.) route every 12 h starting 24 h prior to infection until the end of the experiment. Mice were anesthetized using isoflurane and challenged with SARS-CoV-2 strain MA10 by i.n. instillation (10^5^ PFU in 25 μL) on day 0. At 3 days post-infection (DPI), mice underwent euthanasia and lung tissues were placed in 1 mL PBS with 0.5 mm zirconium oxide beads (Next Advance, Inc. Cat #ZROB05-RNA) and stored at −80 °C for subsequent evaluation by plaque assay. Lung homogenates were spun down at 10,000× *g* for 5 min at 4 °C. Supernatants were aliquoted in 500 μL and stored at −80 °C. 200,000 Vero E6 cells per well were plated on a 12-well plate. The monolayer was washed once with PBS. Serial log_10_ diluted samples (320 μL) were placed in each well. The plate was incubated for 1 h with rocking every 15 min to prevent drying of the cells. After adsorption, 2 mL carboxymethylcellulose overlay (1× MEM, 2% FBS, 2% carboxymethylcellulose) was placed in each well. The plate was incubated at 37 °C for 3 days, after which 4% PFA was added to each well, and cells were stained with 1% crystal violet in 20% ethanol.

The golden Syrian hamster experiment was performed by Dr. Anthony Griffith’s team at the Boston University School of Medicine. Adult hamsters (3 males and 3 females per group) were administered with antiviral compounds i.p. starting 24 h prior to infection until completion of the study. Hamsters were infected with SARS-CoV-2 WA/01 i.n. (4 × 10^4^ PFU in 100 μL). At 4 DPI, hamsters underwent euthanasia and their lungs were collected to perform plaque assays to measure virus titer in the tissues. Lung tissue from each hamster was stored at −80 °C prior to titer. Tissues were removed from −80 °C and thawed at 4 °C before being weighed and cut to a weight ≤100 mg. Each tissue sample was then homogenized using a TissueLyser II (Qiagen, Germantown, MD, USA) in a final volume of 1 mL using DMEM/2% FBS/antibiotic-antimycotic (Gibco^™^). Each tissue homogenate was then diluted and 200 μL of each dilution or control was added to confluent monolayers of Vero E6 cells in duplicate and incubated for 1 h at 37 °C and 5% CO_2_. The plates were gently rocked every 5–10 min to prevent monolayer drying. The monolayers were then overlaid with a 1:1 mixture of 2.5% Avicel^®^ RC-591 microcrystalline cellulose and carboxymethylcellulose sodium (DuPont Nutrition & Biosciences, Wilmington, DE, USA) and 2× MEM (Temin’s modification supplemented with 2× antibiotic-antimycotic (Gibco^™^), 2× GlutaMAX, and 10% FBS (Gibco^™^). Plates were incubated at 37 °C and 5% CO_2_ for 2 days. The monolayers were fixed with 10% neutral buffered formalin and stained with 0.2% aqueous Gentian Violet (RICCA Chemicals, Arlington, TX, USA) in 10% neutral buffered formalin for 30 min, followed by rinsing and plaque counting.

### 2.8. Statistical Analysis

Using a built-in non-linear regression analysis in GraphPad Prism Version 9, 50% inhibitory concentration (IC_50_) and 50% cytotoxicity concentration (CC_50_) values were calculated. All values were calculated by concentration of the inhibitor versus response showing variable slopes using four parameters. Ordinary one-way ANOVA with multiple comparisons was used.

## 3. Results

### 3.1. Amantadine, Rimantadine, and Tromantadine Inhibit SARS-CoV-2 In Vitro in Human Lung Epithelial Cells

**Figure 1.**
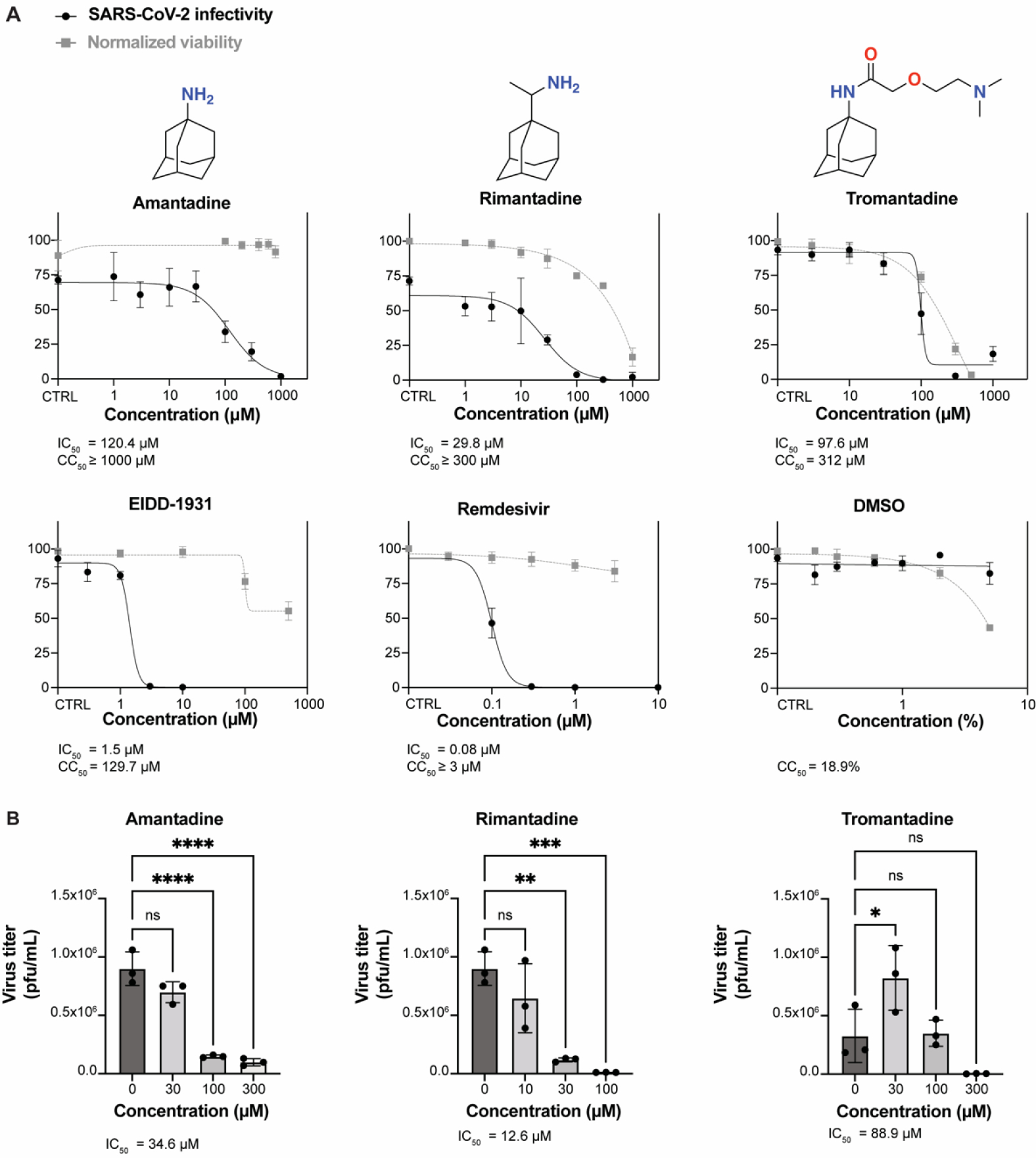
IC_50_ and CC_50_ values for amantadine, rimantadine, and tromantadine against icSARS-CoV-2-mNG in ACE2-A549 cells. (**A**) Cell monolayers were infected with icSARS-CoV-2-mNG with an MOI of 0.1. All drugs remained in media during virus adsorption and until fixation 3 days post-infection. Remdesivir and EIDD-1931 were included as positive controls and dimethyl sulfoxide (DMSO) as a negative control. Forty-eight h later, cells were fixed and were stained with 4′,6-diamidino-2-phenylindole (DAPI); then, fluorescence was quantified. SARS-CoV-2 infectivity (left *y*-axis) is the percentage of FITC-positive cells compared to all DAPI-positive cells. Viability was measured and expressed as percent luminescence relative to the no-drug control (CTRL) (right *y*-axis). Each dot represents technical triplicates and error bars represent median of the triplicates. Each graph is the best representation of two independent experimental replicates. (**B**) Supernatants from SARS-CoV-2-infected cells incubated with each compound were collected and virus was quantified by plaque assay. The error bars indicate the S.D. for three biological replicates of each sample. One-way ANOVA was used to calculate significance. ns, not significant; *, *p* < 0.05; **, *p* < 0.01; ***, *p* < 0.001, ****, *p* < 0.0001.

We assessed the efficacies of amantadine and its derivatives against SARS-CoV-2 infection in vitro by examining growth of infectious-clone-derived mNeonGreen SARS-CoV-2 (icSARS-CoV-2-mNG) in ACE2-A549 cells. Remdesivir and EIDD-1931, the active form of molnupiravir, were included as positive controls for the assay. Figure 1A shows the inhibition of SARS-CoV-2 by amantadine and rimantadine at IC_50_ values of 120 μM and 30 μM, respectively, based on measurement of fluorescence at 3 DPI with a multiplicity of infection (MOI) of 0.1. The CC_50_ values were determined by cell viability assays and were considerably higher than the IC_50_ values for each drug. While tromantadine inhibited SARS-CoV-2 at an IC_50_ of 98 μM, considerable cytotoxicity was also observed (Figure 1A). Supernatants collected at 2 DPI were further used in plaque assays to quantify the virus titer in the presence of different concentrations of the compounds. The virus titers measured by plaque assays corresponded with the fluorescence scoring (Figure 1B).

**Figure 2.**
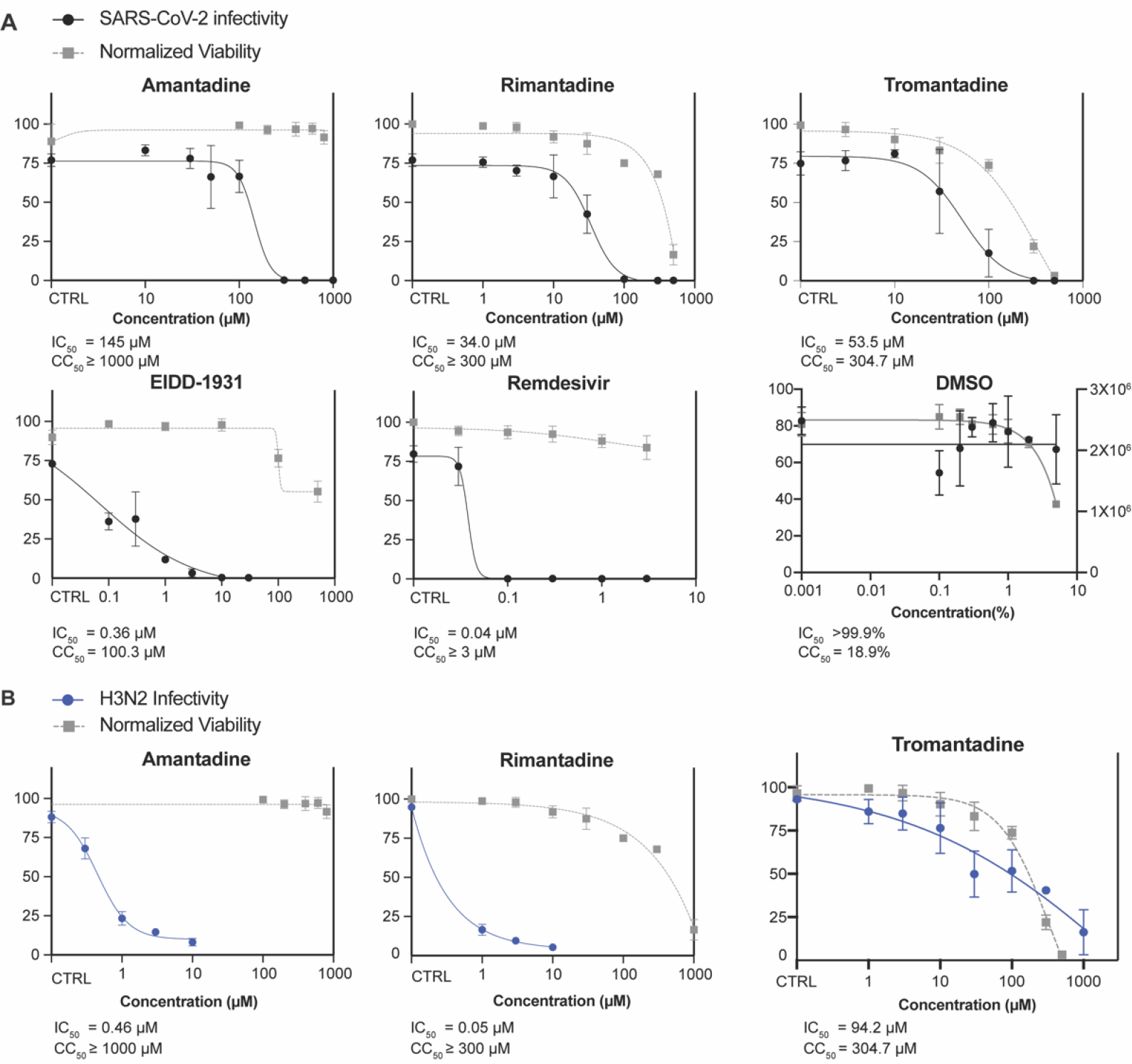
Activity of adamantanes against SARS-CoV-2 WA/01 strain and influenza A virus. (**A**) IC_50_ and CC_50_ values for amantadine, rimantadine, and tromantadine against SARS-CoV-2-WA/01 were determined in ACE2-A549 cells. Remdesivir and EIDD-1931 are included as positive controls. Compound was added during and after virus adsorption, 48 h post-infection cells were fixed and stained with anti-SARS-CoV-2 spike antibody conjugated with AlexaFluor-488 and with DAPI, and fluorescence of individual cells was quantified. SARS-CoV-2 infectivity (left *y*-axis) is the percentage of FITC-positive cells compared to the DAPI-positive cells. Viability is expressed as percent luminescence compared to the no-drug control (CTRL) (right *y*-axis). (**B**). IC_50_ and CC_50_ values for amantadine, rimantadine, and tromantadine were measured against influenza A/Hong Kong/8/68 (H3N2) in ACE2-A549 cells. Forty-eight h after infection and compound incubation, cells were fixed and stained for IAV and fluorescence of individual cells was quantified. IAV infectivity (left *y*-axis) is the percentage of FITC-positive cells compared to the DAPI-positive cells. Viability is expressed as percent luminescence compared to CTRL (right *y*-axis). Each dot represents the median of technical triplicates, and error bars represent the S.D. Each graph shows one of two experimental replicates, each with similar results.

We confirmed each drug’s activity against the WA/01 parental SARS-CoV-2 strain by infecting ACE2-A549 cells with SARS-CoV-2 WA/01 at an MOI of 0.1 and incubating with escalating concentrations of amantadine, rimantadine, or tromantadine. Infection was quantified by staining cells with anti-SARS-CoV-2-spike antibody conjugated with AlexaFluor-488 at 3 DPI. Amantadine, rimantadine, and tromantadine each inhibited SARS-CoV-2 WA/01 in ACE2-A549 cells at IC_50_ values of 136, 34, and 56 μM, respectively, although tromantadine was again toxic to cells (Figure 2A). Influenza A/Hong Kong/8/68 (H3N2) is reportedly sensitive to amantadine [13], so we confirmed the activity of each drug against influenza virus in ACE2-A549 cells using an MOI of 2 and quantifying virus at 3 DPI (Figure 2B). In summary, we concluded that amantadine, rimantadine, and tromantadine exhibit antiviral activity against SARS-CoV-2 in vitro. While amantadine and rimantadine have been shown to similarly inhibit SARS-CoV-2 in vitro in Vero E6 cells, Huh 7.5 cells, and hACE2-A549 cells [14,15], this is the first reported assessment of tromantadine activity against SARS-CoV-2.

### 3.2. Amantadine and Rimantadine Inhibit SARS-CoV-2 Omicron Variant B.1.1.529 In Vitro in Vero E6-TMPRSS2-T2A-ACE2 (Vero E6 T/A) Cells

**Figure 3.**
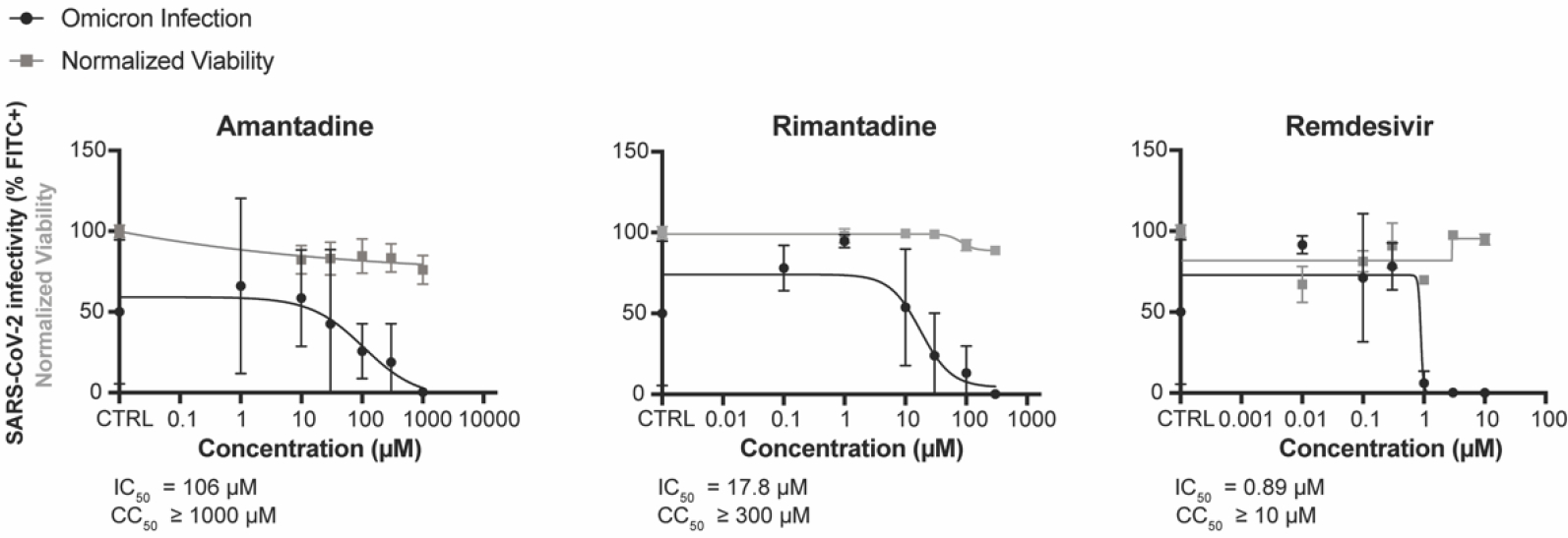
Activity of adamantanes against SARS-CoV-2 omicron variant. Values for amantadine and rimantadine against SARS-CoV-2-WA/01 were determined in Vero E6-TMPRSS2-T2A-ACE2 cells. Remdesivir is included as a positive control. Compounds were added during and after virus adsorption. Forty-eight h after infection, cells were fixed and stained with AlexaFluor-488-conjugated anti-SARS-CoV-2 spike antibody and with DAPI; then fluorescence of individual cells was quantified. Each dot represents the mean of technical triplicates and the error bars indicate the S.E.M.

While ACE2-overexpressed A549 cells represent the SARS-CoV-2 infection site in humans, both Fink et al. and Zhou et al. showed positive efficacies of amantadine or rimantadine in Vero E6 cells, which is generally infectible by SARS-CoV-2 [14,15]. Furthermore, while activity for adamantanes has been tested against the original SARS-CoV-2 strain, it has not been reported against variants of concern such as omicron. To confirm and compare the IC_50_ values of each drug and its efficacy against different variants of SARS-CoV-2, we performed immunostaining assays for Vero E6 T/A cells challenged with the omicron variant of SARS-CoV-2 (Figure 3). The cells seeded in 96-well plates were infected with omicron variant at MOI 0.01 for 48 h and were fixed and stained with mouse anti-SARS-CoV-2 nucleocapsid antibody, followed by staining with goat anti-mouse AlexaFluor-488 and DAPI. Amantadine and rimantadine inhibited SARS-CoV-2 omicron in Vero E6 T/A cells with IC_50_ values of 106 μM and 17.8 μM, respectively. The IC_50_ values are comparable to the IC_50_ values of amantadine and rimantadine obtained by Zhou et al., which were 116 μM and 36 μM [15]. Fink et al. also obtained an IC_50_ value of amantadine of ~83–119 μM [14], further supporting that our assays show accurate inhibitory efficacies of the adamantane drugs against multiple SARS-CoV-2 variants.

### 3.3. *Amantadine and Rimantadine Do Not Significantly Inhibit SARS-CoV-2 Growth in Lungs of* Mice When Administered by Intraperitoneal or Intranasal Route

**Figure 4.**
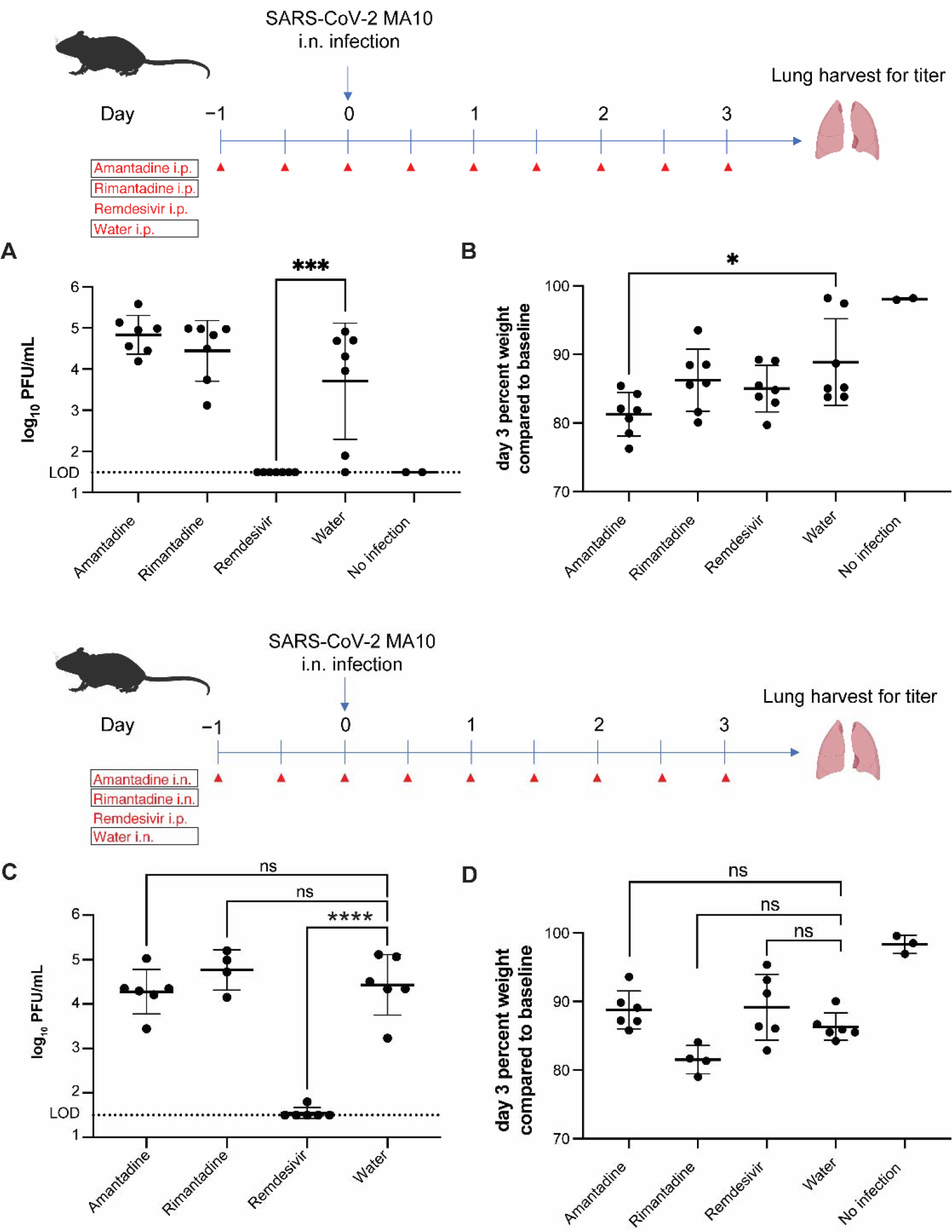
Adamantanes do not inhibit SARS-CoV-2 in BALB/c mouse lungs. (**A**) BALB/c mice (*n* = 7 mice per group) were administered amantadine, rimantadine, remdesivir, or water by intraperitoneal (i.p.) injection twice daily starting 24 h prior to infection with SARS-CoV-2 MA10 intranasally (i.n., 10^5^ PFU per animal). At 3 DPI, mice underwent euthanasia and lung tissues were harvested and homogenized in PBS. Virus titers were determined by plaque assay. Lungs from two uninfected mice were included as negative controls. Each dot represents a value from one animal. One-way ANOVA was used to calculate significance.* *p* < 0.05; *** *p* < 0.001. Data are shown for one of two independent experiments, each with similar results. (**B**) Day 3 weights relative to baseline are shown or each group. (**C**) BALB/c mice (*n* = 6 mice per group) were administered amantadine, rimantadine, or water by i.n. delivery twice daily starting 24 h prior to infection with MA10 SARS-CoV-2 i.n. (10^5^ PFU per animal). Remdesivir was delivered i.p. as for A. At 3 DPI, mice underwent euthanasia and lung tissues were harvested and homogenized in PBS. Virus titer was determined by plaque assay. Two mice in the rimantadine group died within 1 DPI and, therefore, were not available. (**D**) Day 3 weights relative to baseline are shown for each group. Each dot represents a value from one animal. One-way ANOVA was used to calculate significance. ns, not significant; *, *p* <0.05; ***, *p* < 0.001; ****, *p* < 0.0001.

Next, we assessed the anti-SARS-CoV-2 activity of adamantanes in vivo. Since SARS-CoV-2 WA/01 does not infect mice, we opted to use MA10, a mouse-adapted strain of SARS-CoV-2 [16]. Prior to the experiment, we determined the toxicity of each drug in mice based on literature [17] and by administering the highest tolerable dose to mice. We administered 60 mg/kg of amantadine, 60 mg/kg of rimantadine, 50 mg/kg of remdesivir, or water (no-drug control) to four different groups of BALB/c mice (*n* = 7 mice per group) once every 12 h by i.p. injection starting 24 h before infection. Mice were then challenged with 10^5^ PFU of MA10 virus i.n. At 3 DPI, the mice underwent euthanasia and lung tissues were prepared for plaque assay. Mice treated with remdesivir had significantly lower SARS-CoV-2 titers in lung tissues compared to no-drug control mice, while groups injected with either amantadine or rimantadine showed viral titers comparable to water (no-drug control) (Figure 4A). Mice were weighed at baseline and 3 DPI, but the only group to show a significant amount of weight loss compared to water control was that treated with amantadine (Figure 4B). This may reflect systemic toxicity of the drug at this dose.

We tested whether delivery of adamantanes by intranasal route impacted SARS-CoV-2 lung viral titers. We administered 60 mg/kg of amantadine, 60 mg/kg of rimantadine, 50 mg/kg of remdesivir, or water (no-drug control) to four different groups of BALB/c mice (*n* = 6 mice per group) once every 12 h i.n. beginning at 24 h prior to infection with SARS-CoV-2 MA10 and throughout the experiment. Remdesivir control was administered i.p. as described above. Two rimantadine-treated mice were found dead within 1 DPI, which is likely secondary to drug toxicity. Again, lung viral titers were not significantly reduced for either amantadine or rimantadine compared to water control, whereas remdesivir control was effective in reducing lung viral titers (Figure 4C). Mice were weighed at baseline and 3 DPI, and none of the treatment groups had a significant amount of weight loss compared to water control (Figure 4D).

To confirm that the adamantanes have activity against strain MA10 in vitro, we performed experiments in Vero E6 cells since these cells are permissive to MA10 infection. The IC_50_ values were 180 μM for amantadine and 31 μM for rimantadine and were comparable to values with SARS-CoV-2 WA/01 in Vero E6 cells (413 μM and 121 μM, respectively). Thus, adamantanes retain some activity against the SARS-CoV-2 strain MA10.

### 3.4. Rimantadine Inhibits SARS-CoV-2 Growth in Lungs of Hamsters

**Figure 5.**
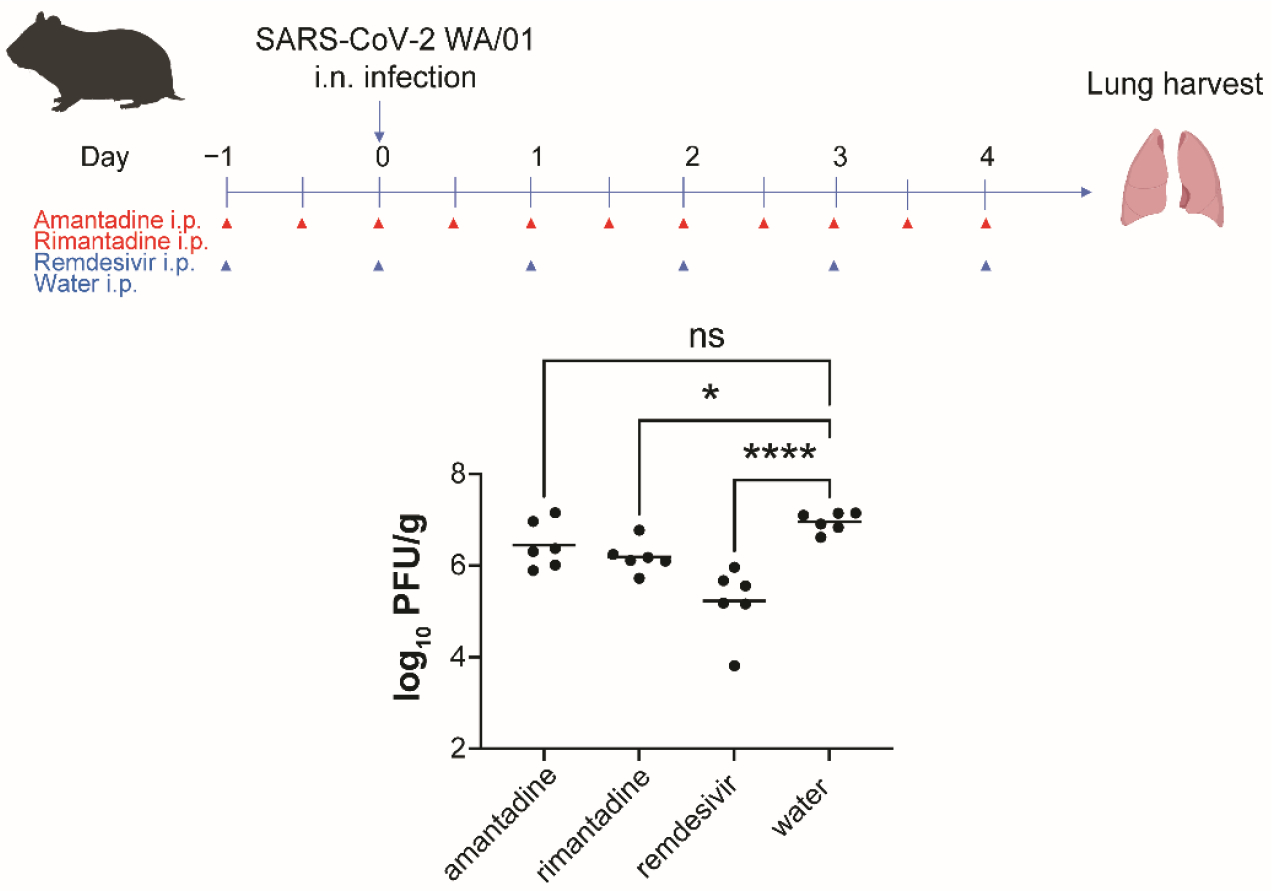
Rimantadine, but not amantadine, shows activity in vivo against SARS-CoV-2 infection of golden Syrian hamsters. Hamsters (*n* = 6 per group) were administered amantadine, rimantadine, remdesivir, or water by i.p. injection starting 24 h prior to infection with SARS-CoV-2 WA/01 i.n. (4 × 10^4^ PFU). At 4 DPI, animals underwent euthanasia and lungs were collected for plaque assays. Each dot represents a value from one animal. One-way ANOVA was used to calculate significance. ns, not significant; *, *p* < 0.05; ****, *p* < 0.0001.

Unlike mice which are not natural hosts of SARS-CoV-2, hamsters serve as a natural model for SARS-CoV-2 infection in the respiratory tract. To establish the efficacies of amantadine and rimantadine in hamsters, we performed an experiment using golden Syrian hamsters based on the drug toxicities observed in mice. Disease in hamsters following SARS-CoV-2 infection peaks at 4 DPI with minimal clinical signs and no discernable differences in weight loss [18–21]. Four groups of hamsters were administered with either 60 mg/kg of amantadine i.p. every 12 h, 60 mg/kg of rimantadine i.p. every 12 h, 15 mg/kg of remdesivir every 24 h, or water i.p. every 24 h starting 24 h prior to infection with SARS-CoV-2 strain WA/01 i.n. (4 × 10^4^ PFU). At 4 DPI, animals underwent euthanasia and lungs were harvested for plaque assays. While amantadine did not significantly inhibit virus compared to no-drug control, rimantadine modestly but significantly inhibited SARS-CoV-2 infection in golden Syrian hamsters (Figure 5).

## 4. Discussion

In this study, adamantanes show varying antiviral efficacies against SARS-CoV-2 in human lung epithelial cells expressing ACE2. Our in vitro assays confirmed antiviral activity of adamantanes against SARS-CoV-2; amantadine, rimantadine, and tromantadine have IC_50_ values of 120–130 μM, 30–40 μM and 60–100 μM, respectively. The IC_50_ values of amantadine, remdesivir, and EIDD-1931 are comparable to published values [14]. Following the in vitro studies, we tested the efficacies of the adamantanes in vivo using BALB/c mice as well as the golden Syrian hamster model. While no antiviral effects were observed with maximal dosages of amantadine or rimantadine by intraperitoneal or by intranasal delivery in BALB/c mice infected with mouse-adapted SARS-CoV-2, rimantadine showed a mild but significant antiviral effect against SARS-CoV-2 strain WA/01 in lungs from infected hamsters. It remains unclear why rimantadine has antiviral activity in hamsters but not in mice; this may be in part due to slightly lower IC_50_ values for rimantadine for SARS-CoV-2 WA/01 (used in hamsters) compared to MA10 (used in mice). Amantadine may fail to show antiviral effects against SARS-CoV-2 due to insufficient bioavailability. The C_max_ plasma level of amantadine in mice following a single-dose i.p. injection of 60 mg/kg is 47 μM, which is less than the IC_50_ of amantadine in vitro for SARS-CoV-2 [22]. Plasma levels of amantadine in mice with i.n. administration or that of rimantadine with i.p. or i.n. administration have not been reported, but the oral administration of 40 mg/kg rimantadine in mice achieves a plasma C_max_ of 9.3 μM, which suggests that the bioavailability of rimantadine may be insufficient for anti-SARS-CoV-2 activity [23]. While our hamster infection study showed a mild antiviral effect of rimantadine with a significant reduction in SARS-CoV-2 replication in lungs, we have not assessed any other endpoints such as lung inflammation or function. Studying additional aspects of animal infection will allow us to identify potential benefits of adamantanes for human COVID-19.

Amantadine may inhibit SARS-CoV-2 by interacting with its ion channels, specifically protein E and ORF10 [24]. Protein E and ORF10 have decreased channel activities of in *X. laevis* oocytes in the presence of either amantadine or hexamethylene-amiloride (HMA), which reportedly interacts with SARS-CoV-2 E protein [24,25]. In response to this study, Harrison et al. confirmed that the E protein seems to function like other model viroporins such as the M2 ion channel of IAV, while other accessory proteins, such as ORF3a, may not meet the viroporin criteria [26]. The nuclear magnetic resonance structure suggests that amantadine, like HMA, binds to the transmembrane domain of the E protein near N-terminal region to disrupt its function. While our studies show that rimantadine inhibits SARS-CoV-2 in vitro and partially in vivo, the channel function of SARS-CoV-2 E protein expressed on the oocytes is not reported to be inhibited by rimantadine [24]. This could be explained by the weaker binding to E protein and suggests that rimantadine could be inhibiting SARS-CoV-2 by interacting with other viral proteins. Understanding how the adamantanes interact with viral proteins including E protein at the molecular level will provide us with strategies to modify the existing compounds to improve their IC_50_ values so that the drugs can be used as effective therapeutics.

The availability of a non-toxic oral drug that could treat or prevent COVID-19, particularly in regions with limited resources, is of importance. The demand for additional agents is likely to increase going forward. Amantadine and rimantadine both have a long record of safety in humans, making them attractive candidates for evaluation for the prophylaxis and treatment for COVID-19. A multiple dose study with the 258 mg extended-release tablet of amantadine once daily reports a C_max_ of 4.16 μM [27], which is considerably lower than that of the in vitro IC_50_ value determined here. The C_max_ for rimantadine ranges from 0.54 μM to 2.17 μM when an adult is given 100 mg for 10 days and is again lower than the IC_50_ value in vitro [15]. A review by Sperber and Hayden noted that blood, salivary, and mucus amantadine concentrations were similar in children with cystic fibrosis. However, the amantadine concentration in autopsy lung tissue from an infant was 14 times the serum concentration. This same review noted that, after single 200-mg doses, the maximum nasal mucus concentrations of rimantadine and amantadine were similar, with the ratio of nasal mucus to plasma concentration about two-fold higher for amantadine [28,29]. It is possible that other formulations of adamantanes could allow for optimized concentrations.

Efficacy of amantadine as an antiviral therapy for COVID-19 in humans remains undetermined; as of now, several human randomized controlled trials addressing adamantanes for COVID-19 are underway (see NCT04952519, NCT04894617, and NCT04854759 [13,30]) and would provide direct evidence of whether adamantanes are effective for human SARS-CoV-2 infection. Both amantadine and rimantadine are associated with neurologic side effects, with rimantadine associated with fewer side effects in both young and elderly adults [31]. Multiple long-term prophylaxis studies in elderly nursing-home patients with rimantadine at 200 mg/day found an excess of adverse reactions [28]. One study concluded that higher plasma concentrations of amantadine seem to correlate with adverse effects, although considerable overlap in concentrations between affected and unaffected participants were noted [28].

If the anti-SARS-CoV-2 activity of amantadine and rimantadine requires doses that are toxic to humans, additive or synergistic combination therapies with non-toxic doses might be effective in the prevention or treatment of COVID-19. Studies of combination therapy with different SARS-CoV-2 antiviral drugs, including adamantanes, will be informative, given that several IAV studies show increased efficacies of amantadine and oseltamivir as combination therapies [32,33]. Known oral SARS-CoV-2 inhibitors such as molnupiravir and nirmatrelvir are candidates for combination therapy alongside the adamantane derivatives. Such an approach could also be effective in preventing the emergence of resistance to single drugs. Finally, additional adamantane derivatives can be tested against SARS-CoV-2 to determine if they have activity against COVID-19.

One important implication of our study is that the adamantane derivatives tested in this study exhibit anti-SARS-CoV-2 activity against variants of concern such as omicron. Studying the mechanism of action of the adamantane derivatives will provide insight for possible therapeutic targets against SARS-CoV-2. Such studies may provide a framework for developing improved antiviral therapies against emerging coronaviruses.

## Data Availability

Not applicable.
